# *Astragalus Mongholicus*: A review of its anti-fibrosis properties

**DOI:** 10.3389/fphar.2022.976561

**Published:** 2022-09-07

**Authors:** Fengying Gong, Rongmei Qu, Yongchun Li, Ying Lv, Jingxing Dai

**Affiliations:** ^1^ Department of Traditional Chinese Medicine, Nanfang Hospital of Southern Medical University, Guangzhou, China; ^2^ Guangdong Provincial Key Laboratory of Medical Biomechanics and Guangdong Engineering Research Center for Translation of Medical 3D Printing Application and National Key Discipline of Human Anatomy, School of Basic Medical Sciences, Southern Medical University, Guangzhou, China

**Keywords:** *Astragalus mongholicus*, fibrosis-related diseases, inflammation, oxidative stress, metabolic regulation

## Abstract

**Background:** Fibrosis-related diseases (FRD) include cerebral fibrosis, pulmonary fibrosis, cardiac fibrosis, liver fibrosis, renal fibrosis, peritoneal fibrosis, etc. The effects of fibrosis can be severe, resulting in organ dysfunction, functional decline, and even organ failure, which can cause serious health problems.

**Aim:** Currently, there is no effective modern medicine for anti-fibrosis in the clinics; however, Chinese medicine has a certain beneficial effect on treating such diseases. *Astragalus Mongholicus* (AM) has rich medicinal value, and its anti-fibrosis effect has been recently investigated. In recent years, more and more experimental studies have been conducted on the intervention of astragaloside IV (AS-IV), astragalus polysaccharide (APS), astragalus flavone, cycloastragalus alcohol, astragalus water extract and other pharmacological components in fibrosis-related diseases, attracting the interest of researchers. We aim to provide ideas for future research by summarizing recent research advances of AM in treating fibrosis-related diseases.

**Methods:** A literature search was conducted from the core collections of electronic databases such as Baidu Literature, Sciencen.com, Google Scholar, PubMed, and Science Direct using the above keywords and the pharmacological and phytochemical details of the plant.

**Results:** AM can be used to intervene in fibrosis-disease progression by regulating inflammation, oxidative stress, the immune system, and metabolism.

**Conclusion:** AS-IV, APS, and astragalus flavone were studied and discussed in detail. These components have high potential anti-fibrosis activity. Overall, this review aims to gain insight into the AM’s role in treating fibro-related diseases.

## Introduction

Fibrosis is a traumatic healing reaction after acute or chronic injury. It can occur in different tissues and organs, such as the brain, lungs, heart, liver, kidneys, intestine, peritoneum, or skin. Fibrosis can develop into severe scarring, and cause abnormalities, weakening of function, and even the failure of organs, which seriously affect human health and quality of life. Studies have indicated that fibrosis-related diseases (FRD) are closely related to inflammation, oxidative stress, fibroblast proliferation, and excessive deposition of the extracellular matrix (ECM) (Wang et al., 2018a).

The researchers turned to traditional Chinese medicine (TCM) because modern medicine has been unable to treat fibrosis effectively. Literature studies have found that most of the drugs used in TCM treatment of FRD are single drugs, monomer components, TCM injections or TCM compounds with the effects of supplementing qi and generating blood, promoting blood circulation and removing blood stasis, while *A. mongholicus* (AM) and its pharmacodynamic components are the focus of relevant research. *Astragalus Mongholicus* is a perennial herb in the genus *A.* of the leguminous family. The dried root of *A. mongholicus Bunge* or/and *Astragalus Amembranaceus (Fisch.) Bge.* was used as medicine (Shuangcheng et al., 2012). AM is a widely used TCM formulation.

**FIGURE 1:**
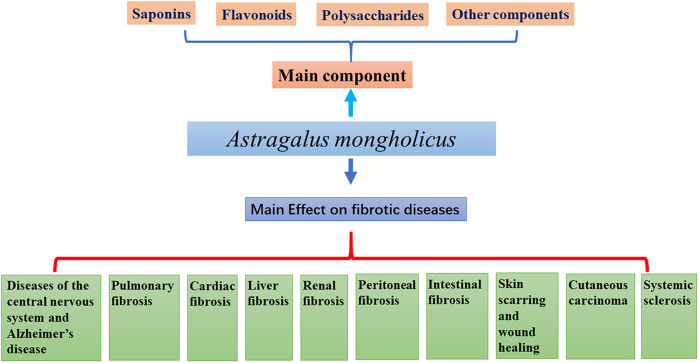
Schematic representation of this review.

AM contains saponins, flavonoids, polysaccharides, and trace elements beneficial to the human body. Combined with modern pharmacological studies, it has been found that AM has anti-tumor properties (Gu et al., 2022) and also, it improves immune function (Liu et al., 2021), protects cardio-cerebrovascular (Li et al., 2022), lung (Qian et al., 2018), kidney (Zhou et al., 2020), liver function (Wang et al., 2022). Besides, it shows an ability to improve intestinal fucntion (Tian et al., 2021), peritoneal function (Li et al., 2014b), and anti-aging ability (Gong et al., 2021). It has also been used for the prevention and treatment of osteoporosis (Kaczmarczyk-Sedlak et al., 2013), antioxidant stress protection (Sheng et al., 2021) and anti-radiation (Wen et al., 2018). This review summarizes the relevant experimental studies on the anti-fibrosis effect of AM (Figure 1).

## Main components of *a. mongholicus*

About 2,000–3,000 species of *Astragalus* are in the legume family (Li et al., 2014a; Podlech, 2008), distributed in the Northern Hemisphere, South America, and Africa. There are ∼278 species of *Astragalus* in China, which are distributed mainly in Tibet (Himalayas), Central Asia, and northeast Asia. AM has important medicinal value; as it is a TCM material used for replenishing qi (Shuangcheng et al., 2012).

More than 200 components have been isolated from *Astragalus* species. The main active components are saponins, flavonoids, and polysaccharides (Ibrahim et al., 2013), but they also contain anthraquinones, alkaloids, amino acids, β-sitosterol, and metal elements (Tan et al., 2020). About 40% of constituent studies have focused on the aboveground parts of AM. Pharmacologic studies have shown that the crude extract of AM and its isolated components have different biological activities: anti-inflammatory, antioxidant, immune stimulation, anti-cancer, anti-diabetes mellitus (DM), heart protection, liver protection, and anti-fibrosis (Wang et al., 2019; Zhao et al., 2012; Zhou et al., 2016).

### Saponins

Saponins are the main chemical constituents of AM. Astragaloside components mainly include Astragaloside I-VIII, Isoastragaloside I, II, and IV, Acetylastragaloside, Cycloastragaloside E, F and G, Agroastragaloside I-IV, and soybean saponin I. Astragaloside is the main active component of saponins, and Astragaloside IV (AS-Ⅳ) is often used as a qualitative and quantitative indices of AM (Ren et al., 2013). AS-Ⅳ shows various activities, such as regulation of calcium balance (Lu et al., 2014; Xu et al., 2008a) as well as an antioxidant (Hu et al., 2009), anti-apoptotic (Liu et al., 2013; Sun et al., 2016), and anti-fibrosis (Hu et al., 2009; Yu et al., 2016) effects.

### Flavonoids

Flavonoid derivatives are one of the main chemical components of AM. Flavonoids include flavonol, flavone, isoflavane, chalcone, flavanol, and thiaxanthin (Berry et al., 1992), and they have antibacterial, antioxidant, and cytotoxic activities, as well as promoting glucose consumption and inhibiting α-glucosidase. The flavonoids of AM have been shown to have an inhibitory effect on tissue fibrosis (Liu et al., 2005; Sun et al., 2007). Phytoestrogen called calycosterone imparts estrogen-like effects by attaching to estrogen receptors and binding to them. Besides being an antioxidant, it has also been reported to have anti-osteoporosis, anti-tumor, and immunomodulatory properties (Song et al., 2017).

### Polysaccharides

There is a type of water-soluble polysaccharide called *Astragalus* polysaccharide (APS), obtained from dried roots or stems of AM. Studies have shown that the monosaccharide components of APS include arabinose, fructose, glucose, and mannose. Xu et al. (Xu et al., 2008b) isolated and purified APS-I and APS-II from the water extract of AM. Kiyohara et al. (Kiyohara et al., 2010) isolated 13 polysaccharides from *A mongolicus*, all showing immunomodulatory activity.

### Other components

AM also contains amino acids, microelements (Li et al., 2014a), sterols, chlorogenic acid (Guzhva, 2010), emodin, and vitamins (Gong et al., 2018a).

## Effect of *a. mongholicus* on fibrotic diseases

### Effect of *a. mongholicus* on diseases of the central nervous system and Alzheimer’s disease

AD is the most common form of senile dementia. The main pathologic features of AD are extensive extracellular amyloid plaques and neurofibrillary tangles (Ramezani et al., 2016; Sperling et al., 2014). Misfolding and aggregation of proteins are linked directly to several neurodegenerative diseases, such as AD, Parkinson’s disease, and Huntington’s disease (Haiyan et al., 2016; Soto and Pritzkow, 2018; Wang et al., 2017). Different types of proteins and peptides misfold and accumulate in the form of filamentous structures that further induce the formation of amyloid fibers. The latter can be found in the extracellular environment around the brain’s arteries and within neurons (Owen et al., 2019; Tahaei Gilan et al., 2019). They cause inflammation and oxidative-stress responses, resulting in decreased neuronal function in the cerebral cortex and hippocampus (Nam et al., 2018).

Song et al. (Song et al., 2017) found that the effects of calycosin on spatial learning and memory were significant and dose-dependent in APP/PS1 transgenic mice (Alzheimer’s disease model). Also, activation of the protein kinase C pathway normalized hippocampal levels of β-amyloid, tau inflammation, oxidative stress, and that neuroprotection depend on it. Wang and Zhao (Wang and Zhao, 2016) reported that Calycosin had a beneficial effect on the improvement, prevention, and treatment of DM-related cognitive deficits by affecting oxidative stress, synaptic function, and the phosphatidylinositol 3-hydroxy kinase/protein kinase B/glycogen synthase kinase-3β (PI3K/AKT/GSK-3β) pathway, and contributed to the improvement of the pathologic process of AD.

#### Effect of the *a. mongholicus* on subarachnoid hemorrhage

The mortality associated with SAH is up to 40%–60%, and SAH is the most dangerous subtype of stroke subtypes (Dong et al., 2018). Chronic hydrocephalus after SAH is closely related to subarachnoid fibrosis. Transforming growth factor β1 (TGF-β1) can promote subarachnoid fibrosis and chronic hydrocephalus by activating the TGF-β1-mothers against decapentaplegic (Smad)-connective tissue growth factor axis to produce different endogenous factors and the ECM (Kuo and Huang, 2021; Yan et al., 2016). SAH can lead to increased malondialdehyde, neuronal apoptosis, caspase-3 rupture, cerebral edema, and decreased superoxide dismutase and glutathione peroxidase activities. AS-IV has been shown to reverse those changes and improve neurobehavioral outcomes in rats with SAH. Those results suggest that AS-IV may alleviate early brain injury after SAH through antioxidant and anti-apoptotic effects (Shao et al., 2014). AS-IV can downregulate the expression of apoptosis-related proteins (forkhead box O1 (*Foxo1*), *Bim*, *Bax*) and inhibit cleavage of caspase-3 through the PI3K/AKT signaling pathway, and alleviate the brain injury caused by SAH (Yang et al., 2020).

#### Effect of *a.s mongholicus* on ischemic stroke

In ischemic stroke, the blood supply is blocked due to the narrowing or blockage of lumina, which results in hypoxia, ischemic necrosis, and loss of nerve function (Lai et al., 2019). Neuroinflammation is involved in almost every step of ischemic brain repair, including neurogenesis (Sun et al., 2020b).

AM, through its antioxidant, anti-inflammatory, and anti-apoptotic properties, has beneficial effects on cognitive impairment after stroke (Xue et al., 2019; L. Zhang et al., 2019b. Zhang et al., 2019a). AS-IV has been found to enhance hippocampal neurogenesis in adults (Huang et al., 2018; Yang et al., 2017) and promote the proliferation of neural stem cells in the brain with transient ischemia (Chen et al., 2019a). Therefore, AS-IV may be a promising strategy for the treatment of ischemic stroke because it promotes neurogenesis (Sun et al., 2020a).

In mice who had suffered a stroke, AS-IV helped inhibit neuronal apoptosis, promote neurogenesis and alleviate cognitive deficits. *In vitro* and *in vivo*, AS-IV can downregulate protein expression of interleukin (IL)-17, antagonize neurogenesis by regulating the AKT/GSK-3β pathway, and significantly regulate apoptosis (Sun et al., 2020b). Calycosin-7-O-β-D-glucoside (CG) was shown to significantly reduce the volume of infarct, the extent of histopathology damage, and the permeability of the blood-brain barrier in a rat model of occluded middle cerebral arteries. CG treatment significantly inhibited the expression and activity of matrix metalloproteinases (MMPs) in the cortical microvessels of ischemic rats and ensured the expression of alveolar protein-1 and tight-junction proteins. CG can also clear nitric oxide (NO), inhibit the activity of MMP-2 and MMP-9, and reduce the death of cultured microvascular endothelial cells of the brain under oxygen-glucose deprivation (OGD) conditions (Fu et al., 2014).

AS-Ⅳ stimulates hippocampal neurogenesis after stroke and downregulates IL-17 expression through the wingless-type (Wnt) pathway, which can promote remodeling and repair of the brain (Sun et al., 2020a). It has been reported that compensatory angiogenesis can occur in the ischemic area after ischemic injury, which somewhat promotes the repair of nerve function in the ischemic area. Studies have shown that AS-IV has a brain-protective effect and is important in promoting angiogenesis. AS-IV has been shown to significantly reduce infarct size, promote cell proliferation and duct formation, and inhibit expression of the ephrinA3 target gene by increasing miRNA-210 expression and inducing activation of hypoxic inducible factor/vascular endothelial growth factor Notch (HIF/VEGF/Notch) signaling pathway (Liang et al., 2020).

AS-IV has a potential neuroprotective in experimental models of Parkinson’s disease, AD, and cerebral ischemia by reducing inflammation and oxidative stress through the antioxidant system (Costa et al., 2019). AS-IV and Calycosin isoflavones may have more important roles in treating cerebral fibrosis.

### Effect of *a. mongholicus* on pulmonary fibrosis

Pulmonary fibrosis is a proliferative disease mainly involving the lung’s interstitium, alveoli, and bronchioles. Gradual loss of alveolar-capillary functional units eventually developed into diffuse pulmonary fibrosis and a “honeycomb” lung. Several types of interstitial lung disease can lead to extensive fibrosis and respiratory failure. A significant proportion of the fibroblasts in pulmonary fibrosis are epithelial cells (Degryse et al., 2011; Tanjore et al., 2009). It has been shown that when epithelial-mesenchymal transformation (EMT) occurs in the lung, epithelial cells show lower levels of E-cadherin while mesenchymal cells have a higher level of a-smooth muscle actin (a-SMA) (Sakuma, 2017). In the development of pulmonary fibrosis, the EMT plays a significant role. The cytokine TGF-β is an important mediator of fibrogenesis. TGF-β1 induces fibroblasts to undergo a phenotypic transition to myofibroblasts, which are the effectors of the fibrotic state (Hinz et al., 2007; Lekkerkerker et al., 2012). Among the potential pro-fibrotic factors known, TGF-α1 is an important one that induces EMT in pulmonary fibrosis (Willis et al., 2005). Therefore, a novel approach employed to treat pulmonary fibrosis could include using a novel anti-EMT pathway or a method that inhibits TGF-β1 signaling to offer a potential target.

#### Effect of *a. mongholicus* on bronchial pulmonary hypoplasia

BPD is a group of heterogeneous pulmonary diseases beginning in the neonatal period. Premature infants suffer from chronic lung injury most commonly caused by barotrauma, volvulus, and oxygen deprivation (Zhang et al., 2022). BPD is characterized by stagnation of lung growth, fewer alveoli, and vascular malformation (Wang and Tsao, 2020). Wang et al. (Wang and Huang, 2014) showed that APS upregulated expression of epidermal growth factor-like domain 7 (EGFL7) and B-cell lymphoma 2, downregulated Bax expression, significantly reduced alveolar injury, and had a protective effect on the lung tissues of BPD patients, which was closely related to inhibition of apoptosis of endothelial cells. Studies conducted on rats with hyperoxia-induced brain damage found that APS has both antioxidant and anti-inflammatory effects.

#### Effect of *a. mongholicus* on idiopathic pulmonary fibrosis

Idiopathic pulmonary fibrosis is the most common type of pulmonary fibrosis. It is characterized by excessive proliferation of fibroblasts/myofibroblasts, excessive deposition of the ECM, alveolar re-epithelialization, and abnormal repair and remodeling of blood vessels (Li et al., 2017). TGF-β1 mediates fibroblast differentiation and plays an important part in pulmonary fibrosis by activating monocytes and fibroblasts through recruitment and increasing ECM production (Wolters et al., 2014). Qian et al. (Qian et al., 2018) found, *in vivo* and *in vitro*, that AS-iV inhibited the FOXO3a hyperphosphorylation induced by TGF-β1/PI3K/AKT, which reversed EMT in the fibrosis process.

### Effect of *a. mongholicus* on cardiac fibrosis

Cardiac fibrosis leads to cardiac dysfunction and arrhythmia in which normal cardiac fibroblasts and circulating fibroblasts proliferate and activate myofibroblasts (Du et al., 2021; Ghosh et al., 2012). Studies have shown that AS-IV has a protective effect on the cardiovascular system. AS-IV can eliminate hypoxia-induced changes, including increased proliferation, decreased apoptosis, and cell death (Lu et al., 2017). Zhang et al. (Zhang et al., 2012) reported that AS-IVcould alleviate hypoxia/reoxygenation-induced cardiomyocyte injury in neonatal rats and that AS-IV may inhibit expression of the long non-coding-RNA growth arrest-specific 5 (GAS5) by activating the PI3K/mammalian target of rapamycin (mTOR) pathway and protect hypoxia-stimulated H9C2 cells (Du et al., 2019). AS-IV may protect cardiomyocytes from hypoxia-induced injury by downregulating the expression of miRNA-23a and miRNA-92a and activating the PI3K/AKT and mitogen-activated protein kinase/extracellular regulated protein kinase (MAPK/ERK) signaling pathways (Gong et al., 2018b). Li et al. (Liang et al., 2017) demonstrated that AS-IV could promote the proliferation of mouse cardiomyocytes and act as cardiac-regenerative cells.

The attenuating effect of AS-IV on myocardial hypertrophy and fibrosis in rats is also closely related to pro-inflammatory signaling pathways. Studies have shown that AS-IV promotes the expression of the suppressor of IKKε (SIKE, an important negative regulator of myocardial hypertrophy) by inhibiting the TANK-binding kinase 1 (TBK1)/PI3K pathway, thereby ablating inflammation and apoptosis (Liu et al., 2018). Wan et al. (2018) found that AS-IV and its active saponin cycloastragalus alcohol could prevent myocardial fibrosis in mice by inhibiting the NLR family pyrin domain containing-3 inflammasome (Wan et al., 2018). AS-Ⅳ protects isoproterenol-induced cardiac hypertrophy by inhibiting the toll-like receptor 4/nuclear factor-kappa B (TLR4/NF-κB) signaling pathway and reducing inflammation (Yang et al., 2013). AS-Ⅳ can prevent (at least in part) isoproterenol-induced hypertrophy and alleviate energy-metabolism disorders through the NF-κB/Peroxisome Proliferator-Activated Receptor-γ Coactivator 1α (PGC-1α) pathway (Zhang et al., 2015b). AS-IV inhibits (at least in part) cardiac hypertrophy by activating the nuclear factor-erythroid 2-related factor 2 (Nrf2)/heme oxygenase-1 (HO-1) pathway (Nie et al., 2019). In one study, Liu and colleagues demonstrated that AS-IV might work as a potential therapeutic for cardiac hypertrophy by inhibiting apoptosis and inflammation and enhancing the action of IKK inhibitors by inhibiting TBK1/PI3K/AKT active pathways (Liu et al., 2018).

The preventive effect of AS-Ⅳ on fibrosis may be related to the TGF-β1-Smad signaling pathway (Chen et al., 2011). Downregulation of the expression of TGF-β1 and its downstream phosphorylated (p)Smad2/3 and p-Smad4 is carried out by AS-Ⅳ, which ultimately decreases the level of type-I collagen (Chen et al., 2021; Wei et al., 2020). In addition, *In vivo* and *in vitro*, AS-IV protects against hypoxia-induced cardiac fibrosis by inhibiting transient receptor potential cationic channel M subfamily member 7 expression (Lu et al., 2017; Wei et al., 2020).

The mechanism by which AS-Ⅳ inhibits isoproterenol-induced myocardial fibrosis may be related to reactive oxygen species (ROS)-mediated responses. Studies have shown that AS-IV can reduce myocardial ROS content and inhibit cardiotrophin-1 expression, ROS-mediated MAPK activation, myocardial-fibroblast proliferation, and collagen production, thereby inhibiting myocardial fibrosis (Dai et al., 2017; Jia et al., 2017; Xu et al., 2016).

In the development of dilated cardiomyopathy, toxic myocardial lipids arising from abnormal lipid metabolism play a significant role. (e.g., myocardial inflammation and fibrosis) which, ultimately, leads to cardiac dysfunction and myocardial remodeling (Kim et al., 2018; Kocabaş et al., 2019). AS-Ⅳ has been shown to improve systolic and diastolic function and histopathologic changes in rats suffering from type-2 diabetes mellitus (T2DM). In addition, Myocardial fibrosis in T2DM rats was inhibited significantly by AS-IV, providing evidence that AS-IV may improve myocardial lipid metabolism in the context of protection from myocardial injury (Wang et al., 2020).

The studies mentioned above suggest that AS-Ⅳ may be an effective therapeutic strategy for preventing myocardial fibrosis.

### Effect of *a. mongholicus* on liver fibrosis

Excessive ECM protein deposition is a hallmark of hepatic fibrosis, which can develop into liver cirrhosis and hepatocellular carcinoma (Friedman, 2008). Most chronic liver diseases are characterized by fibrosis, which shows different developmental patterns depending on the cause of liver damage. As liver fibrosis progresses to cirrhosis, it often leads to the end-stage disease of the organ, so it is vital to limit the progression of chronic liver disease to cirrhosis. Hepatic stellate cell (HSC) activation plays an important part in liver fibrosis (Chen et al., 2019b), and can increase ECM generation by activating the TGF-β1/Smad signaling pathway in HSCs (Yu et al., 2018). Flavonoids have an inhibitory effect on liver fibrosis (An et al., 2021; Cheng et al., 2017; Tan et al., 2006). Network pharmacological analyses have suggested that the mechanism of action of flavonoids against liver fibrosis may be related to inhibition of the NF-κB pathway by inhibition of IKKβ expression (An et al., 2021).

Using porcine serum-induced rats, Liu et al. (Liu et al., 2009) found AS-IV inhibited collagen synthesis in activated HSCs by inhibiting the P38 MAPK pathway mediated by oxidative stress. (Li et al., 2013). Studies have shown that flavonoids can improve dimethylnitrosamine-induced fibrosis through antioxidant activity. The anti-fibrotic activity of flavonoids in chronic liver injury may be due to decreased synthesis of lipid peroxidation (LPO) and collagen and increased collagen degradation (Liu et al., 2005).

An AM extract inhibits fibrosis and adhesion in rats with chronic liver disease through antioxidant activity (Liu et al., 2005). Dang et al. (Dang et al., 2008) found that APS could reduce hepatomegaly and liver fibrosis by decreasing serum levels of alanine aminotransferase and total bilirubin and increasing serum albumin levels. The alleviating effect of AM on chronic liver injury may be related to its ability to enhance antioxidant enzymes. Cheng et al. (Cheng et al., 2017) found that total flavonoids could reduce collagen deposition and regulate the interaction between the peroxisome proliferation-activated receptor-γ signaling pathway and farnesoid X receptors to achieve an anti-fibrosis effect. AS-Ⅳ had an anti-fibrosis effect on porcine-serum-induced rats, possibly inhibiting collagen synthesis and proliferation of hematopoietic stem cells (Liu et al., 2009).

Compound *Astragalus* and *Salvia miltiorrhiza* extract (CASE) inhibits diethylnitrosamine-induced hepatocellular carcinoma by reducing the expression of pre-tumor markers (gamma-glutamyl transferase and glutathione S-transferase placental type (GST-P) and reducing fibrosis severity. CASE may ameliorate liver fibrosis by reducing plasminogen activator inhibitor-1 (PAI-1) mRNA transcription in hepatocellular carcinoma (Rui et al., 2014). *Paeonia lactiflora* and *A. Mongholicus* extract (PAE) has an anti-fibrosis effect on PS-induced rats, and the mechanism of action may be related to PAE scavenging free radicals, reducing platelet-derived growth factor receptor beta (PDGFR-β) expression, as well as inhibiting HSC proliferation and MAPK activation (Sun et al., 2012).

### Effect of *a. mongholicus* on renal fibrosis

Renal fibrosis is common pathogenesis of chronic kidney disease that ultimately leads to end-stage renal failure. Ureteral obstruction and DM can induce interstitial infiltration of inflammatory cells into the kidney, apoptosis of renal tubular epithelial cells, myofibroblast accumulation, promote the production of pro-fibrotic factors, increase ECM production, and reduce ECM degradation. Those actions lead to the development of renal interstitial fibrosis and damage to renal function (Che et al., 2015; Tan et al., 2006).

#### Effect of *a. mongholicus* on renal interstitial fibrosis

An important factor contributing to renal interstitial fibrosis is the deposition of excessive amounts of ECM through the infiltration of interstitial inflammatory cells, the release of pro-inflammatory mediators, and the activation and proliferation of interstitial cells. (Zhou et al., 2017). After the renal injury, the number of interstitial lymphocytes, macrophages, and interstitial collagen fibers increases. A tubular interstitial injury is a key event in the progression of chronic kidney disease because it leads to fibrosis, tubular atrophy, and interstitial peritubular capillary occlusion, thereby resulting in persistent disorders of renal hemodynamics. Renal tubular epithelial cells are the targets of injury and repair of the kidney (Zhou et al., 2017). These cells secrete different pro-inflammatory cytokines and the ECM after inflammatory stimulation. AS-Ⅳ has been shown to prevent renal fibrosis caused by unilateral ureteral obstruction by reducing the inflammatory response *via* the TLR4/NF-κB pathway. EMT induces the progression of renal tubular interstitial fibrosis. TGF-β1 is a well-characterized pro-fibrotic cytokine associated with renal disease and plays a key part in EMT (Hills and Squires, 2011; Matsuda et al., 2011; Qi et al., 2008; Shan et al., 2016; Suzuki et al., 2004).

AM antagonizes the EMT and accumulation of ECM in renal tubules by the TGF-β/Smad2/3 pathway, thereby improving renal fibrosis. Che et al. (2015) found that AS-Ⅳ regulated the activity of MAPK and NF-κB signaling pathways in a dose-dependent manner, and inhibited TGF-β1-induced proliferation, trans-differentiation and ECM formation of fibroblasts. AM can inhibit renal interstitial fibrosis *in vivo*, which may be related to inhibiting myofibroblast activation, inducing hepatocyte growth factor (HGF) expression, and inhibiting TGF-β1 expression (Zuo et al., 2009). AS-IV alleviates the progression of renal fibrosis by inhibiting the MAPK and TGF-β/Smad signaling pathways (Wang et al., 2014).

AS-Ⅳ and Ferulic acid (the main component of *Astragalus* and Angelica) synergistic inhibition of obstructive nephropathy renal tubular interstitial fibrosis in rats, and this inhibition of mesenchymal into renal tubular epithelial - EMT and fibroblast activation, and increase the production of NO in the kidney (Meng et al., 2011). A decoction combined with AM and *Angelica sinensis* had an anti-fibrosis effect on rats with chronic kidney disease caused by ureteral obstruction and could improve renal blood flow in rats with acute ischemic injury to the kidney. The mechanism of action may be related to the activation of endothelial nitric oxide synthase and ROS clearance, promoting NO production (Meng et al., 2007; Wojcikowski et al., 2010).

#### Effect of *a. mongholicus* on diabetic nephropathy

DN is one of the most common causes of end-stage renal disease, and its pathogenesis involves mesangial dilation, thickening of the basement membrane, glomerular hypertrophy, and renal fibrosis, among which progressive renal fibrosis is an important pathologic feature of DN (Chen et al., 2019b). DN’s development depends on oxidative stress and inflammation (Kandhare et al., 2017; Zheng et al., 2016).

Zhang et al. (2020) used a rat model of DN to explore the transcriptome characteristics of the kidney after AS-IV treatment: the latter could significantly reduce the level of advanced glycation end-products, IL-1β expression, and IL-18 expression in the serum and kidney of rats, and the Renal Fibrosis Index. Studies have shown that AS-IV reduces high glucose-stimulated renal tubular EMT through the mammalian target of rapamycin complex 1 (mTORC1)/ribosomal protein S6 kinaseβ-1 (p70S6K) signaling pathway and downregulates the expression of the transcription factors SNAIL and TWIST in HK-2 cells (Chen et al., 2019b). AS-Ⅳ treatment has been shown to improve endoplasmic reticulum stress in renal tubular epithelial cells and renal function and fibrosis in animal models of DN (Ju et al., 2019; Liu et al., 2017; Wang et al., 2018b; Wang et al., 2018c). Wu et al.(2018) pointed out the anti-fibrosis effect of total flavonoids *in vivo* and *in vitro*, and discussed the inhibitory effect of total flavonoids on renal fibrosis through the miRNA-21/Smad7 signaling pathway.

### Effect of *a. mongholicus* on peritoneal fibrosis

Peritoneal dialysis (PD) and hemodialysis are treatment options for patients with end-stage renal disease. However, PD predisposes to encapsulated peritoneal sclerosis, a severe disease with a high mortality rate that can lead to failure of ultrafiltration and eventual discontinuation of treatment (Jagirdar et al., 2019). AM can inhibit the recruitment and activation of monocytes/macrophages, thereby reducing TGF-β1 production in the peritoneum of people undergoing dialysis. Peritoneal fibrosis has been shown to be reduced significantly after AM treatment. Expression of Monocyte Chemoattractant Protein (MCP)-1 was positively correlated with TGF-β1 sensitivity, suggesting that the anti-fibrosis effect of AM is related to McP-1 and TGF-β1 pathways (Li et al., 2014b). AM might be an efficacious agent against PD-induced peritoneal fibrosis. In a study by Zhang et al. (Zhang et al., 2015a), AS-IV was demonstrated to have the therapeutic ability to regulate peritoneal fibrosis by promoting upregulation of Smad7, a signaling component involved in TGF- β1/Smad signaling.

### Effect of *a. mongholicus* on intestinal fibrosis

Intestinal fibrosis is a major complication of Crohn’s disease. Celiac disease is a difficult disease to treat. Liu et al. (Liu et al., 2019) found that Calycoflavones inhibited the expression of p-Smad2, p-Smad3, Smad4, and TGF-β1, inhibited the TGF-β/Smad pathway, and increased Smad7 expression. Therefore, Calycoflavones may inhibit intestinal fibrosis by inhibiting the TGF-β/Smad pathway.

### Effect of *a.s mongholicus* on skin scarring and wound healing

A keloid is a benign skin tumor caused by the proliferation of fibroblasts and capillary endothelial cells after skin injury (Ogawa, 2022). Chen et al. (Chen et al., 2012,^2013^) showed that *Astragalus* armour glycosides of scar have shown significant dose-dependent inhibition; its mechanism is to reduce fibroblasts' secrete collagen type I/III and TGF-β1 levels.

### Effect of *a. mongholicus* on cutaneous carcinoma

Exposure to ultraviolet-B radiation initiates and progresses the generation of squamous-cell carcinoma (Bowden, 2004). More than 70% of all skin-cancer cases in older people are nonmelanoma skin cancer, which is thought to be caused by excess exposure to ultraviolet light accumulated over time (Leiter et al., 2014). From the perspective of preventing photoaging and/or potential skin cancer, AM extract significantly reduces ultraviolet A-induced DNA damage in cultured lung and skin fibroblasts from humans (Curnow et al., 2016).

### Effect of *a. mongholicus* on systemic sclerosis

SSc is a group of diseases of a complex multi-organ system of unknown etiology. Internal organs and skin fibrosis result from excessive fibroblast proliferation and ECM production in this disease. (Qi et al., 2014; Yasuoka et al., 2014). Fibrosis is a major cause of morbidity and mortality in SSc (Rimar et al., 2014). Studies have suggested that targeted fibrosis therapy may be an option for SSc (Gerber et al., 2013). The application of AS-IV consistently suppressed collagen and fibronectin production in the skin lesions of SSc mice with skin lesions and reduced collagen formation and structure. The reduction of AS-IV-induced fibrosis may be due to the deregulation of Smad 3/Fli-1, the major mediators of the fibrotic response and key molecules in TGF-β signaling. AS-IV can also reduce the p-SMAD3 level and completely block its relocation into nuclei. AS-IV attenuates fibrosis by inhibiting the TGF-β–Smads3 axis in SSc(Qi et al., 2014).

## Clinical study

It is difficult for clinicians to identify the differences in clinical efficacy and safety of TCM. AM, a commonly used traditional Chinese medicine, has important medicinal value. The research community has done a lot of research on the mechanism of astragalus membranaceus against fibrosis-related diseases and achieved considerable experimental results, but the clinical application of AM drug preparations is relatively insufficient.

### *A. mongholicus* preparation

At moderate doses, AM granules were sufficient to show the best effect in improving cardiac contraction. In improving the quality of life of patients with chronic heart failure, the effectiveness of AM granules showed a dose-dependent trend (Yang et al., 2011). A prospective randomized controlled study showed that conventional drugs plus AM capsules improved left ventricular diastolic function in hypertensive women with postmenopausal metabolic syndrome (Li et al., 2018). Tian et al.(2016) demonstrated that AM injection or AM water decoction can be used as adjuvant therapy for T2DM based on a preliminary meta-analysis. *Astragalus* is a traditional Chinese medicine used to treat strokes. PG2 is an insoluble polysaccharide extracted from *Astragalus* membranaceus. Li et al. (2018) studied the effect of PG2 on patients with spontaneous acute cerebral hemorrhage. The results showed that PG2 administration for 2 weeks did not increase the percentage of Glasgow Outcome Scale (4-5 scores) and Good Mrs Scores (0–2 scores), nor did it produce any anti-inflammatory properties.

### Traditional chinese medicine compound

Wang et al. (2021) found that various effective components of AM decoction jointly act on liver fibrosis with a comprehensive strategy based on pharmacokinetics. Twenty-four prototype components and 17 metabolites in AM decoction were identified *in vivo*, and the pharmacokinetic characteristics of 14 components were elucidated. Among these components, AS-IV, CAG, glycyrrhizic acid, glycyrrhetinic acid, liquiritigenin, and isoliquiritigenin decreased the expression of α-SMA mRNA. CAG, CG, formononetin, glycyrrhetinic acid, liquiritin, and isoliquiritin restrained the expression of type I collagen. Calycosin, liquiritigenin, isoliquiritigenin, CAG, and glycyrrhetinic increased the apoptosis of human hepatic stellate. The multicomponent combination of AM decoction reduced serum transaminase activity and liver collagen fiber deposition in CCL4-induced liver fibrosis mice. Cheng et al.(2019) found that CDC42 and GLI1 might be the treatment target of AM decoction granules in patients with hepatitis B cirrhosis. In addition, SMAD2, EGFR, AKT1, Rho A, and GAS5 may be related to the efficacy of AM decoction granules in patients with hepatitis B cirrhosis.

A pilot study showed that CD25 expression on T cells was significantly increased after 24 h of echinacea combined with AM and licorice examined CD25 expression on T cells after the ingestion of three common herbs (echinacea, AM, and licorice) alone and in combination (Zwickey et al., 2007). Using AM, angelica Sinensis, rhubarb, and salvia miltiorrhiza, the risk of end-stage renal disease and death in patients associated with advanced chronic kidney disease was decreased. And his benefit did not increase the risk of hyperkalemia.

## Summary and prospects

AM contains flavonoids, saponins, polysaccharides, and other active ingredients. These ingredients have pharmacologic effects: regulating immunity, protecting the cardiovascular and nervous systems; anti-tumor effects; liver protection; multi-target and multi-pathway anti-fibrosis actions; slowing down the progress of fibrosis and alleviating related symptoms. The main action mechanisms of AM are antioxidant, anti-inflammatory, and immunoregulatory, and their combination.

Scholars have conducted a wide range of pharmacologic studies on AM and its active components and explained its mechanism of action at whole-cell, molecular, and genetic levels. However, most of those studies have been experimental and focused on a single ingredient (Table 1). Few clinical studies have been done, so AM has not been applied clinically.

**TABLE 1:** Studies on single ingredients of *Astragalus mongholicus*.

No	Research object	Model construction	Pharmaceutical ingredients	Results and mechanism	References
	**Brain fibrosis**				
1	Male SD rats (280–350 g, n = 68)	SAH	AS-IV	AS-IV inhibited lipid peroxidation, stimulated GSH-Px activity, increased SOD activity, alleviated EBI after SAH through antioxidative and anti-apoptotic effects	Shao et al. (2014)
2	Male SD rats	SAH	AS-Ⅳ	A protective effect of AS-IV in SAH-induced brain injury through regulating PI3K and NF-κB signaling pathways	Yang et al. (2020)
3	Experimental group: male APP/PS1 transgenic mice (n = 60) control group: male C57BL/6 mice (n = 36)	Dimethyl sulfoxide	Calycosin	Calycosin moderated oxidative stress and inflammatory responses in the hippocampus of Alzheimer’s-disease mice by triggering the protein kinase-C pathway and enhancing cognitive function	Song et al. (2017)
4	Male SD rats (n = 40)	Streptozotocin	Calycosin	Calycosin had a valuable effect on the amelioration, prevention, and treatment of DM-associated cognitive deficits through oxidative stress, synaptic function, and the PI3K/Akt/GSK-3β pathway	Wang and Zhao, (2016)
5	Eight-week-old SD rats (n = 18) Primary hippocampus cells	Uninduced NSCs; AS-IV-induced NSC transplant	AS-IV	AS-IV improved the learning and memory of AD rats by encouraging the proliferation and differentiation of NSCs partially through the Notch signaling pathway	Haiyan et al. (2016)
6	Male ICR mice (n = 60)	Oligomeric Aβ	AS-IV	AS-IV could improve oligomeric Aβ-induced cognitive impairment, neuroinflammation, and neuronal damage by decreasing microglial activation and protein expression of NADPH oxidase	Chen et al. (2021)
7	APPswe/PSEN1dE9 double-transgenic mice (n = 40) and C57BL/6 WT mice Transfected SH-SY5Y cells with pEGFP-N1-BACE1	GW9662	AS-IV	AS-IV activated PPARγ and inhibited Aβ production that BACE1	Wang et al. (2017)
8	C57BL/6 mice IL-17 KO mice primary hippocampal NSCs	Photochemical brain ischemia	AS-IV	AS-IV stimulated hippocampal neurogenesis after stroke by helping the brain to remodel and mend with decreasing IL-17 expression by the Wnt pathway	Sun et al. (2020b)
9	Adult male SD rats	Occlusion of the middle cerebral artery	Calycosin and CAG	CAG shielded BBB completeness in cerebral ischemia–reperfusion injury by regulating the NO/cav-1/MMPs pathway	Fu et al. (2014)
10	Male adult SD rats survived after surgery (n = 72) HUVECs	Occlusion of the middle cerebral artery; endostatin	AS-IV	AS-IV activated the HIF/VEGF/Notch signaling pathway through miRNA-210 to encourage angiogenesis and brain protection after ischemic injury to the brain	Liang et al. (2020)
11	Pregnant mice (n = 5) WT mice (n = 93) IL-17 KO mice (n = 32)	Photochemical brain ischemia	AS-IV	IL-17 level was decreased by AS-IV *in vivo* and *in vitro*, and AS-IV had an antagonistic effect on neurogenesis by regulating the AKT/GSK-3β pathway and decreasing apoptosis	Sun et al. (2020b)
	**Myocardial fibrosis**				
1	Male 6-week-old C57BL6 mice (n = 60) Primary cardiomyocytes isolated from 1-2-day-old mice	Aortic banding surgery	AS-IV	AST-IV prevented cardiac hypertrophy by restraining TBK1/PI3K/AKT activity and increasing SIKE expression	Liu et al. (2018)
2	Male SD rats (200 ± 20 g; n = 40)	T2DM	AS-IV	AS-IV protected T2DM-induced myocardial injury in rats by enhancement of lipid metabolism in cardiomyocytes	Wang et al. (2020)
3	Adult rats with chronic heart failure (n = 50) H9C2 cells	Chronic heart failure; angiotensin II	AS-IV	AS-IV constrained cardiac hypertrophy by activating the Nrf2/HO-1 pathway	Nie et al. (2019)
4	Male adult SD rats (220–250 g) Second or third generations of neonatal CFs	Isoproterenol	AS-IV	ASG constrained cardiac fibrosis by targeting the miR-135a-TRPM7-TGF-β/Smad pathway	Wei et al. (2020)

5	SD rat pups (1–3 days; 7 ± 2 g; 40 male, 40 female) Primary cultures of CFs	Isoprenaline	AS-IV	AS-IV constrained cardiac fibrosis by inhibiting ROS-mediated MAPK activation	Dai et al. (2017)
6	Healthy 10-week-old male BALB/c mice (24–25 g, n = 30) Primary cardiac fibroblasts of 2-day-old neonatal SD rats	Isoproterenol	AS-IV and CAG	CAG and AS-IV constrained cardiac-fibrosis effects by inhibiting the NLRP3 inflammasome pathway	Wan et al. (2018)
7	Neonatal rat cardiac fibroblasts; NIH-3T3 cells	Hypoxia	AS-IV	AS-IV constrained hypoxia-induced cardiac fibrosis *in vivo* and *in vitro* by reducing TRPM7 expression	Lu et al. (2017)
8	Male SD rats (180–200 g, n = 30); Primary cardiac fibroblasts of 1-3-day-old SD rats	Isoprenaline	AS-IV	AS-IV inhibited isoprenaline-induced proliferation of cardiac fibroblasts and collagen production through downregulation of ROS-mediated CT-1 upregulation	Jia et al. (2017)
9	SD rats Primary cardiac fibroblasts	Isoprenaline	AS-IV	AS-IV inhibited isoprenaline-induced cardiac fibrosis by constraining ROS-mediated MAPK activation	Dai et al. (2017)
10	Six-week-old male SD rats (200–250 g, n = 40)	Isoproterenol	AS-IV	AS-IV inhibited isoproterenol-induced vascular dysfunction by constraining eNOS uncoupling-mediated oxidative stress and ROS/NF-κB pathways	Xu et al. (2016)
11	Six-week-old male SD rats (180–200 g; n = 30) Ventricular myocytes of neonatal rats	Isoproterenol	AS-IV	AS-IV inhibited isoproterenol-induced hypertrophy and constrained energy-metabolism disorders partially by the NF-κB/PGC-1α pathway	Zhang et al. (2015a)
12	SD rats Cardiomyocytes from neonatal rats	Isoproterenol	AS-IV	AS-IV inhibited isoproterenol-induced myocardial hypertrophy by constraining the TLR4/NF-кB pathway and attenuating the inflammatory effect	Yang et al. (2013)
13	BALB/c mice	Coxsackievirus B3	AS-IV	AS-IV inhibited myocardial fibrosis of coxsackievirusB3-induced dilated cardiomyopathy by decreasing expression of the TGF-β1-Smad pathway	Chen et al. (2011)
	**Pulmonary fibrosis**				
1	Rats (n = 40) Human type-II alveolar epithelial cells (A549)	Bleomycin to rats TGF-β1 to A549 cells	AS-IV	AS-IV inhibited EMT in bleomycin-induced pulmonary fibrosis by increasing expression of FOXO3a and the TGF-β1/PI3K/AKT pathway	Qian et al. (2018)
2	Adult female SD rats (n = 80)	Bleomycin	AS-IV	AS-IV inhibited lung fibrosis by decreasing HMGB1 release and ECM production	Li et al. (2017)
3	Newborn SD rats (n = 96) and fungible mother rats	Hyperoxia	APS	APS reduced airway remodeling and alveolar damage by increasing EGFL7 expression and exerting protective effects against BPD in neonatal rats	(X. H. Wang et al., 2014)
	**Liver fibrosis**				
1	HSC-T6 cells	TGF-β1	Formononetin	Flavonoids antagonized liver fibrosis by suppressing the NF-κB pathway by constraining of IKKβ expression	An et al. (2021)
			Isorhamnetin		
			Kaempferol		
			Calycosin		
2	HSC-T6 cells	Platelet-derived growth factor-BB	AS-IV	AM constrained hepatic fibrosis and cirrhosis *via* HSC senescence and apoptosis by promoting the NF-κB pathway	Chen et al. (2019c)
3	Male SD rats (150 ± 10 g) (n = 53)	Dimethylnitrosamine	astragalus flavone	TFA inhibited fibrosis by regulating the PPARγ pathway and interaction with farnesoid X receptors	Cheng et al. (2017)
4	Male SD rats (180–200 g) HepG2 cells	Dimethylnitrosamine	CASE	CASE inhibited dimethylnitrosamine-induced hepatocarcinogenesis by suppressing fibrosis and mRNA transcription of PAI-1	Rui et al. (2014)
5	Male Wistar rats (120–140 g) Primary hepatic fibrosis	Porcine serum	PAE	PAE antagonized fibrosis induced by porcine serum by obliterating free radicals, reducing expression of PDGFR-β, and suppressing HSC proliferation and MAPK activation	Sun et al. (2012)
6	Male Wistar rats (130–150 g; n = 50) Primary HSCs	Porcine serum	AS-IV	AS-IV inhibited the fibrosis induced by porcine serum *via* its inhibitory effects on the synthesis and proliferation of collagen in HSCs	Liu et al. (2009)
7	Male SD rats (130–150 g) (n = 32) Primary HSCs	Carbon tetrachloride	PAE	PAE-inhibited hepatic fibrosis might be associated with its ability to scavenge free radicals, reduce the level of TGF-β1, and inhibit the synthesis and proliferation of collagen in HSCs	Sun et al. (2007)
	**Intestinal/peritoneal fibrosis**				
1	HMrSV5 cells	TGF-β1	AS-IV	AS-IV activated upregulation of Smad7 in the TGF-β1/Smad pathway during the EMT of HMrSV5 cells	Zhang et al. (2015b)
2	Male SD rats (180–200 g)	Standard PD fluid	AM	AM suppressed the recruitment and activation of monocytes/macrophages and decreased the TGF-β1 level in dialyzed peritoneal membranes	Li et al. (2014b)
3	Human intestinal fibroblasts	TGF-β1	Calycosin	Calycosin constrained intestinal fibrosis by suppressing the TGF-β/Smad pathway	Liu et al. (2019)
	**Renal fibrosis**				
1	Male SD rats (n = 16) HK-2 cells	High-fat-diet and streptozotocin injection to induce DM	AS-IV	AS-IV treatment significantly decreased serum and kidney levels of AGEs, IL-1β, and IL-18 and fibrosis indices of rats	Zhang et al. (2020)
2	HK-2 cells	High glucose	AS-IV	AS-IV suppressed high glucose-induced renal tubular EMT by blocking the mTORC1/p70S6K pathway and consequent reduction of expression of the transcription factors SNAIL and TWIST in HK-2 cells	Chen et al. (2019b)
3	Male C57BL6 mice (n = 20) HK-2 cells	Unilateral ureteral obstruction; lipopolysaccharide (100 ng/ml)	AS-IV	AS-IV antagonized progression of renal fibrosis by suppressing inflammation via the TLR4/NF-кB pathway	Zhou et al. (2017)
4	Male C57BL6 mice (n = 30) NRK-52E cells	Unilateral ureteral obstruction; lipopolysaccharide (100 ng/ml)	AM	AM inhibited tubular EMT by suppressing the Smad pathway	Shan et al. (2016)
5	Primary renal fibroblasts isolated from 5-6-week-old BALB/c mice	TGF-β1	AS-IV	AS-IV improved renal interstitial fibrosis by regulating MAPK and NF-kB pathways	Che et al. (2015)
6	SD rats (n = 48)	Unilateral ureteral obstruction	AM	The renal protective effect of AM might be related to inhibition of myofibroblast activation, induction of HGF, and reduction of TGF-β1 expression	Zuo et al. (2009)
7	Male SPF Wistar rats NRK-49F cells HK-2 cells	Unilateral ureteral obstruction TGF-β1-induced NRK-49F cells and interleukin-1-induced HK-2 cells	AS-IV and FA	AS-IV and FA suppressed renal tubulointerstitial fibrosis by constraining tubular EMT, fibroblast activation, and promoting NO production in the kidney	Meng et al. (2011)
	**Skin scarring, skin cancer, systemic sclerosis**				
1	Female eight-week-old SD rats (200–250 g; n = 12)	Skin excision	AS-IV	AS-IV increased wound re-epithelization, angiogenesis, and regulated ECM remodeling by activating the proliferation and migration of keratinocytes, promoting new-vessel formation, and balancing the synthesis and disposition of collagen	Chen et al. (2012)
2	Six-week-old SD rats (n = 24) New Zealand rabbits (n = 6) Human skin fibroblasts	Full-skin excision Skin-irritation test of rabbits	AS-IV	AS-IV showed an angiogenetic effect on wound repair and an inhibitory effect on scar complications accompanied by healing in adult tissues	Chen et al. (2013)
3	Lung fibroblasts from human fetuses Human skin fibroblasts	UVA + visible light	Marshmallow and AM	Hydroponically grown root extracts from AM and marshmallow decreased UVA-induced DNA damage in lung and skin fibroblasts by suppressing oxidative stress	Curnow et al. (2016)
4	Treated or untreated systemic sclerosis and normal fibroblasts 6–8-week-old female C57/BL6 mice (n = 18)	Bleomycin	AS-IV	AS-IV attenuated fibrosis by inhibiting the TGF-β–Smads3 axis in systemic sclerosis	Qi et al. (2014)

## Glossary

AD: Alzheimer’s disease;
AKT: protein kinase B
AM: *Astragalus mongholicus*
APS: *Astragalus* polysaccharide
AS-IV: astragaloside IV
BPD: bronchopulmonary dysplasia
CASE: compound *Astragalus* and Salvia *miltiorrhiza* extract
CAG: cycloastragalus alcohol
CG: calycosin-7-O-β-D-glucoside
DM: diabetes mellitus
DN: diabetic nephropathy
ECM: extracellular matrix
EGFL7: epidermal growth factor-like domain 7
EMT: epithelial–mesenchymal transformation
ERK: extracellular regulated protein kinase
FRD: fibrosis-related diseases
GAS5: growth arrest specific 5
GSK-3β: glycogen synthase kinase-3β
GST-P: glutathione S-traferase placental type
HGF: hepatocyte growth factor
HIF: hypoxic inducible factor
HO-1: heme oxygenase-1
HSCs: hepatic stellate cells
IL-17: interleukin
LPO: lipid peroxidation
MAPK: mitogen-activated protein kinase
MCP-1: Monocyte Chemoattractant Protein
MMP: matrix metalloproteinase
mTORC1: mammalian target of rapamycin complex 1
NF-κB: nuclear factor-kappa B
Nrf2: nuclear factor-erythroid 2-related factor 2
OGD: oxygen-glucose deprivation
p70S6K: ribosomal protein S6 kinaseβ-1
PAE: Paeonia lactiflora and *Astragalus Mongholicus* extract
PAI-1: plasminogen activatorinhibitor-1
PD: peritoneal dialysis
PDGFR-β: platelet-derived growth factor receptor beta
PGC-1α: peroxisome proliferator-activated receptor-γ coactivator 1α
PI3K: phosphatidylinositol 3-hydroxy kinase
PS: porcine-serum
SAH: subarachnoid hemorrhage
SIKE: The suppressor of IKKε
SSc: systemic sclerosis
T2DM: type-2 diabetes mellitus
TBK1: TANK-binding kinase 1
TCM: traditional Chinese medicine
TGF-β1: transforming growth factor β1
TLR4: toll-like receptor 4
